# Changes in Pulmonary Hypertension Following Transcatheter Aortic Valve Implantation: Implications for Prognosis

**DOI:** 10.3390/jcm14103463

**Published:** 2025-05-15

**Authors:** Hao-Wei Lee, Chi-Hung Huang, Chih-Hui Chin, Po-Chin Chou, Chia-Hsiu Chang, Eng-Thiam Ong

**Affiliations:** 1 General Cardiology, Cardiovascular Center, Cathay General Hospital, Taipei 106, Taiwan; 2 School of Medicine, College of Medicine, National Yang Ming Chiao Tung University, Taipei 112, Taiwan; 3 Cardiovascular Center, Cathay General Hospital, Taipei 106214, Taiwan; 4 School of Medicine, National Tsing Hua University, Hsinchu City 300, Taiwan; 5 School of Medicine, College of Medicine, Fu Jen Catholic University, New Taipei 242, Taiwan; 6 Interventional Cardiology, Cardiovascular Center, Cathay General Hospital, Taipei 106, Taiwan

**Keywords:** pulmonary hypertension, transcatheter aortic valve implantation, severe aortic stenosis

## Abstract

**Background:** Pulmonary hypertension (PH) is a known prognostic factor in the setting of transcatheter aortic valve implantation (TAVI), but data on post-TAVI PH changes and their clinical relevance are limited. **Method:** This retrospective cohort included 55 PH patients (52.7% male; mean age 81.5 ± 8.9 years) classified by pre-TAVI systolic pulmonary artery pressure into mild (36–50 mmHg), moderate (50–60 mmHg), and severe (≥60 mmHg) PH. PH was reassessed using the closest post-TAVI echocardiogram. The primary outcome was a 2-year composite of all-cause mortality or heart failure hospitalization. **Result:** PH improved in 61.8% and worsened in 14.5% of patients after TAVI. Those with worsened PH had a significantly higher risk of adverse outcomes compared to those with no PH change (log-rank *p* = 0.029), while event rates were similar between improved and unchanged PH groups (log-rank *p* = 0.742). Cox regression analysis identified PH worsening as an independent predictor of adverse outcomes (HR: 8.775; 95% CI: 2.669–28.851; *p* < 0.001). **Conclusions:** PH deterioration after TAVI appears to be associated with worse outcomes, possibly more indicative than PH improvement.

## 1. Introduction

Pulmonary hypertension (PH) is commonly observed in patients with severe aortic stenosis (AS) and serves as an important prognostic factor following transcatheter aortic valve implantation (TAVI) [1,2]. Studies have shown that the presence of PH before TAVI is associated with reduced survival, and the severity of PH further influences clinical outcomes, with higher pulmonary pressures correlating with worse prognosis [3,4]. However, TAVI has been demonstrated to lower pulmonary pressure relatively soon after the procedure [3,4,5,6]. A reduction in systolic pulmonary artery pressure (sPAP) following TAVI is linked to improved survival rates [5,6], whereas residual PH is often associated with poorer clinical outcomes [3,4,6]. Despite these findings, inconsistencies in PH classification, particularly in defining cutoff values, complicate result interpretation across studies. While some studies examine PH’s impact on TAVI outcomes, comprehensive research on post-TAVI pulmonary pressure dynamics and their clinical significance remains limited. Notably, the effects of worsening PH on patient outcomes are rarely explored. Therefore, this study aims to investigate changes in PH after TAVI in patients with severe AS and assess their impact on clinical prognosis.

## 2. Materials and Methods

### 2.1. Participants

Between September 2016 and February 2025, patients diagnosed with severe AS who received TAVI at Cathay General Hospital in Taiwan were consecutively included in this study. The definition of severe AS followed established guidelines, characterized by one or more of the following criteria: a mean pressure gradient across the aortic valve of at least 40 mmHg, a peak aortic jet velocity equal to or exceeding 4 m per second, or an aortic valve area less than 1.0 cm^2^ (or an indexed area below 0.6 cm^2^/m^2^) [7,8]. The decision to proceed with TAVI was made following a thorough evaluation and consensus by the multidisciplinary heart team. The selection of prosthesis type and size was determined by the local heart team, guided by pre-procedural assessments including echocardiography and multidetector computed tomography. The choice of TAVR access route was made at the discretion of each heart team. Patients were excluded if the latest echocardiography before TAVI showed no evidence of PH or if a post-TAVI echocardiography was not performed. PH was defined as sPAP ≥ 36 mmHg [9,10].

### 2.2. Study Design

This is a retrospective, single-center cohort study. Detailed clinical information was obtained by reviewing patient medical records. The study protocol received approval from the Institutional Review Board of Cathay General Hospital (approval ID: CGH-P113040) and adhered to the ethical standards set forth in the Declaration of Helsinki. All enrolled patients underwent a thorough transthoracic echocardiographic assessment prior to TAVI, and received post-procedural follow-up in line with guideline recommendations [11,12]. For each echocardiographic study, both image acquisition and Doppler measurements were performed and verified by two echocardiography specialists. During retrospective analysis and PH staging, every echocardiogram was independently assessed and validated by two cardiologists blinded to clinical data and patient outcomes. sPAP was evaluated by transthoracic echocardiography and calculated by adding the peak velocity of tricuspid regurgitation (TR) to the estimated right atrial pressure (RAP), following the principles of the modified Bernoulli equation [10,11]. The RAP was estimated based on measurements of the inferior vena cava (IVC) size and its changes with respiration as follows: a value of 3 mmHg was assigned when the IVC diameter < 2.1 cm with inspiratory collapses > 50%, whereas an IVC diameter > 2.1 cm that collapses by < 50% led to an estimated RAP of 15 mmHg. When the IVC diameter and its degree of collapse fell outside the above criteria, a default value of 8 mmHg was applied [11,13]. Before TAVI, patients were classified into three groups according to sPAP level: mild PH (sPAP ≥ 36 mmHg and < 50 mmHg), moderate PH (sPAP ≥ 50 mmHg and < 60 mmHg), and severe PH (sPAP ≥ 60 mmHg). After TAVI, PH resolution was defined as sPAP < 36 mmHg. PH improvement was defined as a decrease by at least one severity category compared with baseline (e.g., from severe or moderate to a lower category, or to resolved). PH worsening was defined as an increase by at least one category compared with baseline (e.g., from mild or moderate to a higher category). We then examined the association between the PH change and clinical outcomes, adjusting for other relevant clinical factors, including right ventricular (RV) dysfunction at baseline and residual severe PH after TVAI. RV dysfunction is defined as meeting any one of the following criteria: tricuspid annular plane systolic excursion < 1.7 cm, S′ < 9.5 cm/s, or fractional area change < 35% [10].

### 2.3. Outcomes

The primary outcome of this study was defined as a combination of all-cause mortality or hospitalization resulting from heart failure (HF) within a follow-up period of up to two years.

### 2.4. Statistical Analysis

Statistical analyses were conducted using SPSS software (version 21.0; IBM Corp., Armonk, NY, USA). Data are presented either as mean values accompanied by standard deviations or as counts with corresponding percentages. For categorical variables, comparisons were made utilizing the chi-square test. Continuous variables following a normal distribution were analyzed between groups using the independent (unpaired) Student *t*-test, whereas non-normally distributed data were assessed with the Mann–Whitney U test. The primary outcome was evaluated through event-free survival analysis employing Kaplan–Meier curves, and differences were tested using the log-rank method. To identify independent predictors of the primary outcome, Cox proportional hazard regression was performed. Variables demonstrating a *p*-value of less than 0.10 in univariate analysis were incorporated into a multivariable regression model. Adjusted hazard ratios (HRs) along with 95% confidence intervals (CIs) were calculated after controlling for possible confounders. A two-tailed *p*-value below 0.05 was considered indicative of statistical significance.

## 3. Results

### 3.1. Baseline Characteristics of Study Population

**Figure 1:**
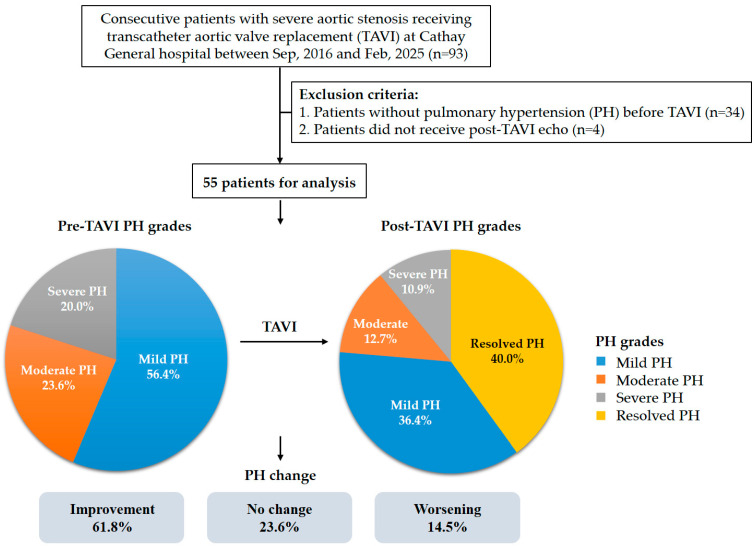
Flow chart of the study.

**Figure 2:**
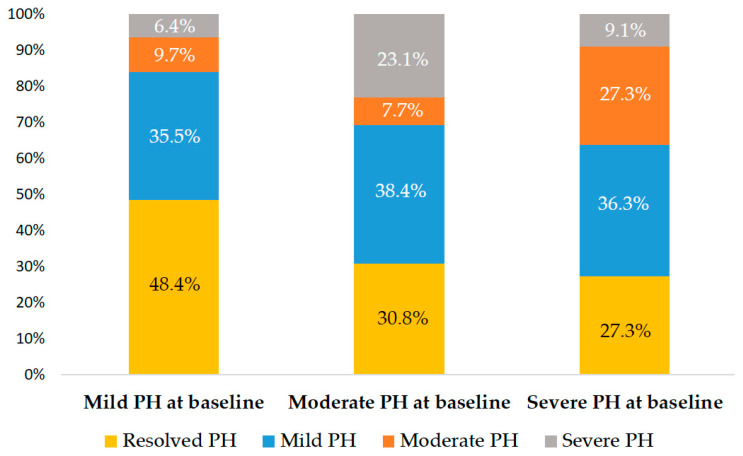
Post-TAVI PH stage distribution. Each bar indicates the proportion of corresponding post-procedural stage based on baseline PH classification.

A total of 93 patients underwent TAVI at Cathay General Hospital during the study timeframe. Following application of the exclusion criteria, 38 individuals were omitted from the analysis. As a result, the final study cohort consisted of 55 eligible patients (Figure 1). The average age of the included participants was 81.5 years with a standard deviation of 8.9 years, and males accounted for 52.7% of the group. A mean STS risk score of 10.8 ± 7.9% indicated a high surgical risk within the population. The average sPAP among the patients was 49.5 ± 10.6 mmHg. A total of 51 patients (92.7%) received TAVI via trans-femoral access and 4 patients (7.3%) via nontrans-femoral access. Furthermore, 46 patients (83.6%) received a self-expanding valve, and 9 patients (16.4%) received a balloon-expandable valve. Other baseline characteristics of the patients are listed on Table 1. At baseline, 31 patients (56.4%) were classified as having mild PH, 14 (25.5%) as moderate PH, and 10 (18.2%) as severe PH. Across the groups, STS risk scores increased with PH severity, while AV peak velocity varied significantly without a consistent trend (Table 1 and Figure 1). The median interval between TAVI and echocardiographic follow-up was 4 days (interquartile range: 3–14 days). After TAVI, 22 patients (40.0%) showed resolution of PH, while 20 (36.4%) had mild PH, 7 (12.7%) had moderate PH, and 6 (10.9%) had severe PH. Compared with baseline, 34 patients (61.8%) experienced improvement in PH, 8 (14.5%) showed worsening, and the remaining patients exhibited no change (Figure 1). Figure 2 shows the distribution of PH grades after TAVI according to baseline PH classification.

### 3.2. Outcomes

**Figure 3:**
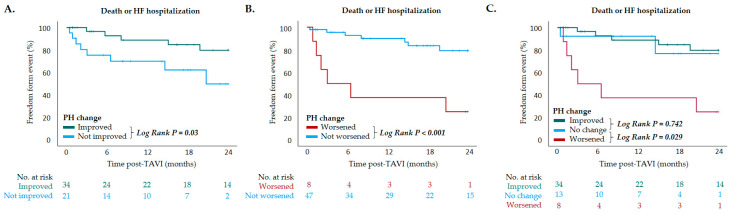
Kaplan–Meier survival curves depicting outcomes of death or heart failure hospitalization in patients with (**A**) PH improved after TAVI vs. PH not improved after TAVI (*p* = 0.03, log-rank test); (**B**) PH worsened after TAVI vs. PH not worsened after TAVI (*p* < 0.001, log-rank test); (**C**) PH improved after TAVI vs. PH not changed after TAVI (*p* = 0.742, log-rank test) and PH worsened after TAVI vs. PH not changed after TAVI (*p* = 0.029, log-rank test). Pulmonary hypertension (PH); TAVI, transcatheter aortic valve implantation.

During a median follow-up of 1.2 ± 0.8 years, 13 (23.6%) patients met the primary composite endpoint of death or HF hospitalization, which included 9 (16.4%) deaths and 4 (7.3%) HF hospitalizations. KM survival analysis revealed that patients exhibiting PH improvement following TAVI experienced a significantly lower rate of mortality or HF hospitalization compared to those without such improvement (log-rank *p* = 0.03) (Figure 3A). In contrast, patients with PH deterioration had a significantly higher risk of adverse events compared to those without worsening (log-rank *p* < 0.001) (Figure 3B). When stratified by changes in PH following TAVI—categorized as PH improvement, no change, or worsening—those with PH worsening exhibited a significantly increased risk of mortality or HF hospitalization compared to patients with no change (log-rank *p* = 0.029) (Figure 3C). Conversely, outcomes were comparable between patients with PH improvement and those with unchanged PH status (log-rank *p* = 0.742) (Figure 3C).

In univariate analyses, older age and PH worsening were linked to an increased risk of the combined endpoint of mortality and HF hospitalization (HR: 1.125; 95% CI: 1.023–1.236; *p* = 0.015 and HR: 6.360; 95% CI: 2.124–19.041; *p* < 0.001, respectively). Variables including age, CKD, and PH worsening were entered into the multivariate model. PH worsening after TAVI remained an independent predictor of adverse outcomes (HR: 8.775; 95% CI: 2.699–28.851; *p* < 0.001). Additionally, older age and CKD were also independently associated with increased risk (HR: 1.116; 95% CI: 1.025–1.216; *p* = 0.012 and HR: 5.128; 95% CI: 1.072–24.530; *p* = 0.041, respectively) (Table 2).

## 4. Discussion

The key findings of this study included the following: (1) TAVI effectively alters sPAP in patients with severe AS within a relatively short timeframe; (2) patients who experienced an improvement in PH after TAVI had a better prognosis than those who did not; and (3) deterioration of PH post-TAVI was strongly associated with a higher risk of adverse clinical outcomes and may serve as an even stronger prognostic indicator than PH improvement.

In patients with severe AS, the presence of PH prior to TAVI is a known predictor of poor prognosis. Higher baseline sPAP levels are associated with more advanced disease and are linked to worse clinical outcomes after the procedure [3,4]. AS contributes to the development of PH primarily through elevated left ventricular (LV) filling pressures, which may be further exacerbated by concurrent mitral regurgitation, diastolic dysfunction, and myocardial hypertrophy [4,13,14]. This leads to increased left atrial pressure and pulmonary venous congestion, ultimately triggering pulmonary vasoconstriction and arterial remodeling [3,4,13,15]. TAVI has been shown to reduce pulmonary blood pressure shortly after the procedure [3,4,5,6], and the resolution of PH after TAVI may lead to a better prognosis. A retrospective study involving 1872 patients from the OCEAN-TAVI (Optimized Transcatheter Valvular Intervention–Transcatheter Aortic Valve Implantation) registry suggested that patients with resolved PH after TAVI had comparable rates of mortality and HF hospitalization after 2 years to those without PH at baseline [6]. However, even without complete resolution of PH, an improvement in its severity may still contribute to better outcomes. Alushi et al. classified patients into three categories based on sPAP: normal (<34 mmHg), mild-to-moderate (34 to <46 mmHg), and severe (≥46 mmHg). They found that patients who demonstrated a reduction in PH grade after TAVI had a lower risk of mortality [5]. Nevertheless, in a retrospective study involving 990 patients by Testa et al., a decrease in sPAP of 15 mmHg or more one month after TAVI did not lead to improved survival [4].

The inconsistency in the cutoff values used to classify PH grades across studies has hindered the standardized application and validation of PH grade changes in relation to clinical outcomes [3,4,5,6]. In our study, PH was defined as an sPAP > 36 mmHg, which corresponds to a tricuspid regurgitation velocity of >2.8 m/s. This cutoff value is well established for the diagnosis of PH according to both American and European guidelines [9,10], and has been validated in previous studies [6,16,17]. In addition, an sPAP ≥ 60 mmHg was used in our study to define severe PH. This threshold has been adopted in earlier research and has demonstrated an association with poor prognosis. Furthermore, persistent PH with an sPAP ≥ 60 mmHg following TAVI has been identified as a predictor of increased mortality. Sinning et al. reported that patients with an sPAP ≥ 60 mmHg three months after TAVI had a significantly higher two-year mortality compared to those with an sPAP < 60 mmHg (50.0% vs. 18.6%; *p* = 0.001) [3]. Similarly, Testa et al. observed elevated one-year mortality in patients with persistent severe PH one month after TAVI (HR: 2.4, 95% CI: 1.5–2.8; *p* = 0.004) [4]. In our cohort, we further stratified PH into three categories using an additional median sPAP cutoff of 50 mmHg, in order to better illustrate the dynamic changes in pulmonary pressure following TAVI and their prognostic implications. We believe that this classification approach not only aligns with existing evidence regarding the prognostic relevance of PH severity, but also provides a more refined assessment of how post-TAVI changes in sPAP may impact patient outcomes. Using this grading system, our study found that although patients with PH improvement after TAVI appeared to have better clinical outcomes, which is consistent with previous studies [5,6], further stratification of those without PH improvement into unchanged and worsened PH groups revealed no significant prognostic difference between the improved and unchanged PH groups. The key contribution of our study is highlighting the impact of PH worsening after TAVI, a topic rarely addressed in previous reports. The mechanisms behind PH development after TAVI remain unclear. Since LV remodeling is slow and only partially reversible, early post-TAVI changes are more likely to be due to the acute reduction in LV afterload and filling pressure rather than structural remodeling [18,19]. In cases of longstanding PH, irreversible pulmonary vascular changes may have occurred, requiring longer time to reverse. Thus, worsened PH after TAVI may reflect underlying advanced pulmonary vascular pathology [5]. Our findings demonstrate that post-TAVI deterioration in PH is strongly associated with poor outcomes and may be a stronger prognostic indicator than PH improvement. Additionally, as TAVI may not fully reverse LV myocardial damage [20], this further underscores the essential role of adjunctive medical therapy in optimizing outcomes for patients undergoing TAVI. Sodium-glucose cotransporter-2 inhibitors have been reported to be associated with significant improvement in LVEF and a marked decrease in sPAP [21]. Future studies are warranted to investigate the impact of pharmacological therapies on cardiac remodeling after TAVI.

This study has several limitations. First, it was a single-center retrospective study with a small population, and further research with larger sample sizes is needed to validate our results. Second, right heart catheterization (RHC) is considered the gold standard for assessing sPAP [22,23], while we used echocardiographic measurement due to its wide availability, noninvasiveness, and cost-effectiveness. However, studies have validated this method, showing adequate correlation with RHC-derived sPAP [24,25]. Third, we did not evaluate outcomes based on the underlying etiology of PH. Previous studies have shown that patients with combined post- and pre-capillary PH have poorer survival following TAVI, whereas those with isolated postcapillary PH experience outcomes comparable to patients without PH [26,27]. Future research investigating PH changes using hemodynamic definitions may help identify which PH subgroups derive the greatest benefit from TAVI in the setting of severe AS. Finally, the inclusion of three covariates in the multivariable Cox regression may have introduced overfitting bias in the context of a limited event count. Consequently, these results should be cautiously interpreted, pending confirmation in studies with greater statistical power.

## 5. Conclusions

TAVI can lead to changes in PH among patients with severe AS over a short period. An early worsening of PH following TAVI appears to be associated with a higher risk of the composite outcome of death or HF hospitalization, and might be a more significant predictor of prognosis than PH improvement.

## Figures and Tables

**Table 1:** Baseline characteristics of all patients and according to PH grades.

Baseline Characteristics	All (*n* = 55)	Mild PH (*n* = 31)	Moderate PH (*n* = 13)	Severe PH (*n* = 11)	*p*-Value
Age, years	81.5 ± 8.9	80.8 ± 8.9	81.8 ± 8.5	83.1 ± 10.0	0.760
Male, *n* (%)	29 (52.7%)	18 (58.1%)	5 (38.5%)	6 (54.5%)	0.489
Body mass index, kg/m^2^	24.0 ± 3.5	23.8 ± 3.7	24.5 ± 4.0	23.9 ± 2.7	0.868
Hypertension, *n* (%)	45 (81.8%)	26 (83.9%)	10 (76.9%)	89 (81.8%)	0.862
Diabetes mellitus, *n* (%)	27 (49.1%)	18 (58.1%)	5 (38.5%)	4 (36.4%)	0.317
Heart failure, *n* (%)	32 (58.2%)	15 (48.4%)	9 (69.2%)	8 (72.2%)	0.243
Atrial fibrillation, *n* (%)	21 (38.2%)	9 (29.0%)	6 (46.2%)	6 (54.5%)	0.260
Coronary artery disease, *n* (%)	29 (52.7%)	15 (48.4%)	9 (69.2%)	5 (45.5%)	0.389
Cerebrovascular disease, *n* (%)	5 (9.1%)	3 (9.7%)	2 (15.4%)	0 (0.0%)	0.420
PAOD, *n* (%)	13 (23.6%)	9 (29.0%)	1 (7.7%)	3 (27.3%)	0.299
Chronic kidney disease, *n* (%)	31 (56.4%)	16 (51.6%)	9 (69.2%)	6 (54.5%)	0.556
Asthma or COPD, *n* (%)	4 (7.3%)	2 (6.5%)	1 (7.7%)	1 (9.1%)	0.957
STS risk score, %	10.8 ± 7.9	8.4 ± 6.4	12.9 ± 9.4	14.8 ± 8.2	0.035
**Echocardiographic findings**					
LVEF, %	55.0 ± 15.6	57.1 ± 15.7	56.2 ± 13.2	47.5 ± 16.9	0.201
AV peak velocity, m/s	4.1 ± 0.9	4.3 ± 0.9	3.6 ± 0.8	3.9 ± 0.8	0.046
AV MPG, mmHg	39.3 ± 18.1	43.4 ± 18.2	32.9 ± 19.2	35.5 ± 14.7	0.159
sPAP, mmHg	49.5 ± 10.6	41.8 ± 3.9	54.3 ± 2.9	65.7 ± 6.8	<0.001
RV dysfunction, *n* (%)	3 (5.5%)	2 (6.5%)	0 (0.0%)	1 (9.1%)	0.579
**Procedural findings**					
Trans-femoral access, *n* (%)	51 (92.7%)	29 (93.5%)	12 (92.3%)	10 (90.9%)	0.957
Nontrans-femoral access, *n* (%)	4 (7.3%)	2 (6.5%)	1 (7.7%)	1 (9.1%)	0.957
Self-expanding valve, *n* (%)	46 (83.6%)	25 (80.6%)	12 (92.3%)	9 (81.8%)	0.624
Balloon-expandable valve, *n* (%)	9 (16.4%)	6 (19.4%)	1 (7.7%)	2 (18.2%)	0.624

AV, aortic valve; COPD, chronic obstructive pulmonary disease; LVEF, left ventricular ejection fraction; MPG, mean pressure gradient; PAOD, peripheral arterial occlusion disease; PH, pulmonary hypertension; RV, right ventricular; sPAP, systolic pulmonary artery pressure; STS, Society of Thoracic Surgeons.

**Table 2:** Univariable and multivariable Cox proportional hazard analysis of mortality or heart failure hospitalization.

	Univariable		Multivariable	
	HR (95% CI)	*p*-Value	HR (95% CI)	*p*-Value
Age, years	1.125 (1.023–1.236)	0.015	1.116 (1.025–1.216)	0.012
Male	0.459 (0.141–1.491)	0.195		
Body mass index, kg/m^2^	0.957 (0.800–1.146)	0.634		
Hypertension	1.786 (0.230–13.848)	0.579		
Diabetes mellitus	1.226 (0.411–3.652)	0.715		
Heart failure	1.241 (0.405–3.802)	0.705		
Atrial fibrillation	1.282 (0.419–3.929)	0.663		
Coronary artery disease	0.968 (0.325–2.882)	0.953		
Cerebrovascular disease	0.796 (0.103–6.151)	0.827		
PAOD	0.637 (0.141–2.876)	0.558		
Chronic kidney disease	4.366 (0.967–19.706)	0.055	5.128 (1.072–24.530)	0.041
COPD	1.111 (0.144–8.577)	0.920		
STS risk score, %	1.036 (0.971–1.106)	0.279		
LVEF, %	1.009 (0.970–1.050)	0.655		
AV peak velocity, m/s	0.800 (0.414–1.546)	0.507		
AV MPG, mmHg	0.996 (0.966–1.027)	0.791		
RV dysfunction	3.463 (0.766–15.699)	0.107		
Nontrans-femoral access	1.315 (0.171–10.127)	0.793		
Self-expanding valve	1.775 (0.226–13.929)	0.585		
Post-TAVI severe PH	2.311 (0.635–8.414)	0.204		
PH worsening	6.360 (2.124–19.041)	<0.001	8.775 (2.669–28.851)	<0.001

AV, aortic valve; CI: confidence interval; COPD, chronic obstructive pulmonary disease; HR, hazard ratio; LVEF, left ventricular ejection fraction; MPG, mean pressure gradient; PAOD, peripheral arterial occlusion disease; PH, pulmonary hypertension; RV, right ventricular; STS, Society of Thoracic Surgeons.

## Data Availability

The data that support the findings of this study are not publicly available due to containing information that could compromise the privacy of the research participants but are available from the corresponding author upon reasonable request.
